# Mesenchymal Stromal Cell Therapy for Thoracic Surgeons: An Update

**DOI:** 10.3390/jpm13121632

**Published:** 2023-11-22

**Authors:** Francesco Petrella, Enrico Mario Cassina, Lidia Libretti, Emanuele Pirondini, Federico Raveglia, Antonio Tuoro

**Affiliations:** Department of Thoracic Surgery, Fondazione IRCCS San Gerardo dei Tintori, 20900 Monza, Italy; enricomario.cassina@irccs-sangerardo.it (E.M.C.); lidia.libretti@irccs-sangerardo.it (L.L.); emanuele.pirondini@irccs-sangerardo.it (E.P.); federico.raveglia@irccs-sangerardo.it (F.R.); antonio.tuoro@irccs-sangerardo.it (A.T.)

**Keywords:** stromal cell, regenerative medicine, drug loading, drug delivery, oncology, thoracic surgery, bronchopleural fistula, pleural mesothelioma, imaging, lipofilling

## Abstract

Stem cells are undifferentiated cells presenting extensive self-renewal features and the ability to differentiate “in vitro” and “in vivo” into a range of lineage cells, like chondrogenic, osteogenic and adipogenic lineages when cultured in specific inducing media. Two major domains of clinical applications of stem cells in thoracic surgery have been investigated: regenerative medicine, which is a section of translational research in tissue engineering focusing on the replacement, renewal or regeneration of cells, tissues and organs to re-establish damaged physiologic functions; drug loading and delivery, representing a new branch proposing stem cells as carriers to provide selected districts with anti-cancer agents for targeted treatments.

## 1. Introduction

Mesenchymal stem/stromal cells (MSC) are undifferentiated cells presenting wide self-renewal features and the capacity to differentiate “in vitro” and “in vivo” into a range of lineage cells, like chondrogenic, osteogenic and adipogenic lineages when cultured in specific inducing media [1,2,3,4,5,6]. There are two primary types of stem cells: (1) embryonic stem cells, coming from the inner cell mass of blastocysts; (2) adult stem cells, which can be isolated from many tissues, like adipose tissue, peripheral blood, bone marrow and, as more recently reported, from some other tissues [7]. Moreover, pluripotent stem cells are a particularly potent type of stem cell that normally only exists during early embryonic development and are able to form all three of the basic body layers (ectoderm/endoderm/mesoderm) and even germ cells; on the contrary, induced pluripotent stem (iPS) cells are a type of pluripotent stem cell deriving from adult somatic cells that have been genetically reprogrammed to an embryonic stem-cell-like state by forcing the expression of genes and factors to maintain the defining properties of embryonic stem cells.

Several studies have spotlighted the promising clinical use of stromal cell properties in many medical areas, including thoracic surgery, but with debatable results [8,9]. Two major domains of clinical applications of stem cells in thoracic surgery have been investigated: (a) regenerative medicine, which is a section of translational research in tissue engineering focusing on the replacement, renewal or regeneration of cells, tissues and organs to re-establish damaged physiologic functions [10]; (b) drug loading and delivery, representing a new branch proposing stem cells as carriers to provide selected districts with anti-cancer agents for targeted treatments [8]. 

## 2. Basic Principles

**Figure 1:**
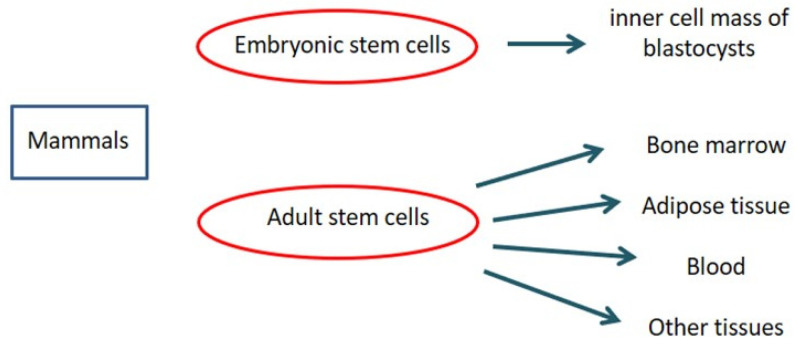
Stem cells in mammals.

Mesenchymal stromal cells (MSC) are undifferentiated multipotent adult cells presenting extensive self-renewal properties and the ability to differentiate “in vitro” and “in vivo” into a wide spectrum of mesenchymal lineage cells [11]. According to the International Society for Cellular Therapy position statement, the minimal criteria to define human MSC are the following: (1) MSC must be plastic-adherent when maintained in standard culture conditions. (2) MSC must express CD105, CD73 and CD90 and lack expression of CD45, CD34, CD14 or CD11b, CD79alpha or CD19 and HLA-DR surface molecules. (3) MSC must differentiate into osteoblasts, adipocytes and chondroblasts in vitro [12]. Thanks to their immune phenotype, MSC can escape the host immune system, thus permitting allogenic transplantation without the need for any immunomodulation [13]: after implantation, in fact, MSC interconnect with the adjacent microenvironment, promoting tissue healing, repair and potential regeneration by cross-talking with a closer type of cells existing within impaired tissues or organs [14]. In addition, MSC show an intense anti-inflammatory and immunomodulatory activity on the immune system by some compounds, mainly chemokines and cytokines [15]. MSC were first observed in bone marrow, and they were presumed to be found only in this district; they were subsequently isolated from many other tissues, both in adult and fetal tissues: placenta, umbilical cord, adipose tissue, dental pulp, tendon, liver, cornea, spleen, thymus, brain, periosteum, synovial and amniotic fluids [7,13] (Figure 1). Although MSC coming from different tissues are different, they show the same profile in terms of secreted cytokines. MSC are capable of moving and implanting at sites of tissue impairment and lesion and at a trauma site, expressing on-site healing activity by the paracrine excretion of anti-inflammatory mediators and wound-healing factors rather than through a trans-differentiation pathway into organ-specific cell types [16,17,18,19,20,21,22,23,24,25].

## 3. Imaging

**Figure 2:**
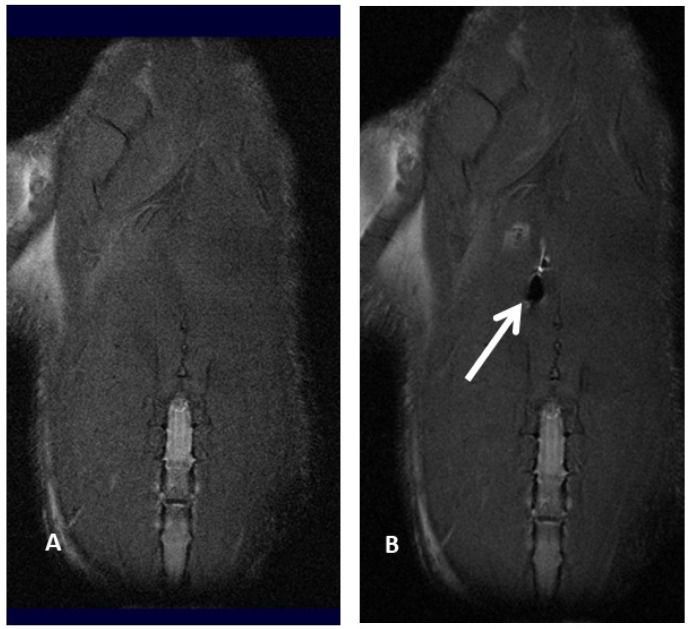
Magnetic resonance imaging of a rat before (**A**) and after (**B**) injection of ferumoxides labeled mesenchymal stromal cell, resulting in an apparent loss of signal (white arrow and black hole).

Molecular imaging provides a better knowledge of cell fate after transplantation, thus optimizing cell therapy results. In fact, it has already been reported that after systemic injection, MSC amass in the lung and vascular beds of other organs and tissues, thus reducing the number of MSC moving to the target site for therapy [26,27]. Positron emission tomography (PET), magnetic resonance imaging (MRI), single-photon emission computed tomography (SPECT), bioluminescence imaging (BLI) and fluorescence imaging (FLI) are several imaging techniques able to visualize signals produced by labeled MSC, thus making it possible to properly track MSC, revealing cell fate, migration and implant after transplantation. Molecular imaging plays a pivotal role in determining the appropriate cell type, transfer technique, cell dosage, and treatment window, as well as in evaluating potential toxic side effects by identifying early cell graft modification into neoplasms, which cannot be detected in a timely manner by standard imaging technique. In addition, molecular imaging of MSC provides useful information about cell survival, proliferation and differentiation within the target area: these data make it possible to optimize the therapeutic dose–response curve, identifying the best dosage and dosing frequency of cell therapy [28,29,30,31,32,33]. To date, imaging and tracking of transplanted MSC have not yet been definitely assessed, and several issues related to MSC migration and engraftment to extra-target sites need to be further assessed before molecular imaging techniques can be safely applied to daily clinical practice. For example, Scharf et coll. have demonstrated that when performing ultrasound-guided MSC intralesional injection for tendonitis, only a few cells remain within collagenous tendon while, on the contrary, many cells scatter to the surrounding fascia, disclosing an MSC greater delocalization than expected [34]. Similarly, extracellular vesicles (EV)—which are paracrine mediators released by MSC under specific conditions, disclosing potential application in cell-based therapies—tend to preferentially migrate to the lung, spleen and liver after systemic administration [35,36,37,38,39,40]. Cellular imaging techniques are not invasive and provide high-resolution images, thus resulting in very important to defining the cell fate after implantation; in experienced hands, they are relatively easy to use, although not widely available (e.g., 8-Tesla MRI for small animals) because of their exclusive use for scientific non-clinical purposes (Figure 2A,B).

### 3.1. Optical Imaging 

This technique—including both bioluminescence imaging and fluorescence imaging—relies on retroviral vectors and enzymes expressing fluorescent proteins. It has been observed that labeled MSC migrated to the liver retained their labels in an in vivo liver regeneration model. These studies disclosed that fluorescence labeling is a promising tool for MSC tracking, in particular for liver diseases [41,42,43,44,45,46,47].

### 3.2. Nuclear Imaging 

Human placental MSC distribution was investigated in nude mice after intravenous injection by carbon radioisotope labeling thymidine [48]. Labeled MSC were found in the spleen, lung, liver, stomach, and left femur of the recipient animal; this observation showed that carbon radioisotope labeling thymidine did not interfere with human placental MSC activity and, therefore, can be considered a valuable option for quantifying the engrafted cells after transplantation in experimental studies [49].

### 3.3. Magnetic Resonance Imaging

Magnetic resonance imaging offers high-resolution images that range from 50 μm in experimental animal models to 300 μm in clinical settings; since it does not rely on ionizing radiations, it is one of the less invasive methods of tracking MSC “in vivo”. However, MSC have to be pre-labeled by some contrast media: among them, there are some positive contrast media, like gadolinium, and some negative contrast media, such as superparamagnetic iron-oxide (SPIO) and ultrasmall superparamagnetic iron-oxide (USPIO), which make it possible to identify and track transplanted MSC within the target environment. Another more recent approach for MSC detection by MRI is MSC labeling by perfluorocarbon (PFC) nanoemulsions, requiring dedicated 19Fluorine MRI. In our personal experience, we compared magnetic resonance imaging of iron-labeled cells and magnetic resonance spectroscopy of fluorine-labeled cells. We explored whether rat bone marrow MSC can be effectively labeled with superparamagnetic iron-oxide (SPIO) nanoparticles and perfluorocarbon (PFC) nanoemulsion formulations without interfering with cell viability and matched magnetic resonance imaging (MRI) and magnetic resonance spectroscopy (MRS) findings from iron-labeled and fluorine-labeled MSC. The SPIO negative contrast enhancement provides an excellent view even in the case of very small numbers of labeled cells but can be easily confused with other sources of magnetic sensitivity effects, like blood vessels or air bleeds, in relation to the anatomical site under evaluation. As prospective clinical applications might include lung tissue evaluation for MSC injection in the repair process and assessing MSC migration and engraftment into the lungs, we also labeled cells with PFC nanoemulsions, which can be identified by 19F MRI. Our finding suggested that both types of contrast agents (PFC and SPIO) can be successfully used for MSC labeling, although further studies were needed to upgrade the efficacy of PFC labeling [47]. In particular, we referred to our previous experience of bronchopleurla fistula (BPF) repair after bronchial disruption following major anatomical lung resection for cancer.

Subsequently, in fact, we investigated the role of 7-Tesla magnetic resonance imaging to track MSC labeled by perfluorocarbon (PFMSC): PFC-based emulsion provided better views in an “in-vivo” rat model compared with USPIO contrast medium, particularly when assessing lung tissue. MSC diffusion to other solid organs, in this case only to the liver, was also better disclosed by PFC-labeled MSC, probably due to the iron content within the liver. A linear decline of delivered PFC-labeled MSC was reported 24 and 48 h after the injection in lung tissue; in nearly half of the observations, PFC-labeled MSC seeding to the liver was reported, with a similar decline over time as observed in the lung. We might argue that MSC locally injected into the lung might scatter to the liver through the blood flow of the cardiac cycle. Nevertheless, considering that about 40% of the rats did not disclose any signal in the liver at time zero, it might be argued that cell migration to the liver only happened when MSC were injected more centrally and thus closer to pulmonary vessels, whereas MSC injected more peripherally in the lung parenchyma were not able to reach the systemic blood flow [50,51].

At the moment, as MSC can be labeled with non-clinical tracers (SPIO, USPIO), there are no authorized methods to track in vivo MSC, and this poses a further issue to be addressed before switching to clinical practice.

### 3.4. Magnetic Particle Imaging

Magnetic particle imaging (MPI) identifies the iron-oxide nanoparticle-labeled MSC by using magnetic fields. It offers an excellent image contrast high sensitivity and provides high-level performance for single-cell tracking, representing a very promising method for tracking transplanted MSC in vivo. It has been demonstrated that MPI cell tracking has a 200-cell detection limit for “in vitro” and “in vivo” monitoring of graft clearance over 87 days in a rat model [52]. MPI successfully demonstrated that transplanted MSC, after intravenous injection, are entrapped in the lungs and then relocated to the liver within one day [53] (Table 1).

## 4. Regenerative Medicine

Regenerative medicine, tissue engineering and stromal cell technologies—combining the specialties of biology and engineering—might offer new therapeutic options for end-stage diseases, thus preventing several major problems related to organ shortage, long-term immunomodulation and chronic rejection [54]. The lungs have significant reparative properties when required in response to particular injuries and damage. One of the most reliable hypotheses is that pulmonary tissue might respond to particular stimuli by triggering off stromal cells or by re-starting the cell cycle to regenerate damaged cells [55,56]. Basal cells can operate as tissue-specific stem cells of the airway epithelium in the proximal airway, while in the distal airway, the bronchiolar epithelium is silent until damage happens when variant club cells—representing a group of secretory cells—disclose the ability to proliferate in response to damage [57,58]. Type II alveolar epithelial cells are judged as the best options for progenitor cells of the adult pulmonary alveola; some of them are able to proliferate, self-renew and generate alveolar type I epithelial cells [59]. At the passage from the bronchiolar region to the alveolar region of the distal airways is the bronchoalveolar duct junction; in this district, there are some club cells—known as bronchoalveolar stem cells—presenting airway epithelial regenerative capacity after induced lung injury. These variant cells are known as bronchoalveolar stem cells, although their existence “in-vivo” has been questioned [3,54]. Three-dimensional cell cultures (3-D) represent the ideal methods to replicate the primary functions of lung tissue, overcoming many limitations of bi-dimensional (2-D) cell cultures, which are not able to fully re-create the “in-vivo” cellular background [60,61,62,63]. Air–fluid interface cell cultures have been created by culturing donor respiratory epithelial cells to reproduce specific diseases like asthma, cystic fibrosis and pollutant-induced airway cell damage, thereby providing more specific data on the underlying pathophysiology [64,65,66,67,68]. In the last few years, human lung organoids have been described as highly effective 3-D “in-vitro” model systems for pulmonary studies on lung cancer, inflammatory diseases and acute infections [69,70,71]. Human lung organoids generated from induced pluripotent stem cells (iPSC) or from adult stem cells allow further investigation of evolutional pathways and the development of personalized medicine approaches [69,70,71]. The recent advances in bioengineering supported the development of “organs on-a-chip”, which are bioengineered devices replicating tissue and organ functions within a controlled environment [72,73,74]. 

## 5. Tissue Engineering

Tissue engineering is a multidisciplinary area utilizing the concepts of biology, medicine and engineering to repair, restore or regenerate damaged organs and tissues. It relies on the isolation and culture of stromal cells, proper scaffolds and an ideal extracellular matrix [75,76,77,78,79,80,81]. In the extremely difficult area of lung and airway regeneration, several cases of bioengineered tracheas have been reported in the past [82,83], albeit with very disappointing results [84,85], and the attractive idea of bioengineered tracheal substitution has not reached a definitive and reliable solution. Xu et al. recently reported their experience regarding long-segment airway defect reconstruction by using an “in-vivo” bioengineered trachea [86]. In their uncontrolled monocentric cohort study, they enrolled three patients presenting extended airway neoplastic lesions involving the main carina. Radical excision of the tumors was achieved using standard surgical procedures. Subsequently, “in-vivo” bioreactor airway reconstruction was realized utilizing a nitinol stent enclosed in two layers of acellularized dermis matrix. Two catheters—connected to external pumps—were located between the layers of the acellularized dermis matrix, and the bioengineered trachea was constantly perfused by antibiotic solution and total peripheral total nucleated cells. In this setting, the patients acted as “in-vivo” bioreactors to provide “in situ” regeneration of the tracheal graft. All the enrolled patients survived the procedure, and long-term bronchoscopy follow-up disclosed well-perfused and regenerated airways without any anastomotic leakages [86]. The aim of pulmonary tissue engineering is to reproduce the entire range of specialized lung tissues, thus providing physiologic activities by a bioengineered airway, vascular tissue and gas exchange tissue [87]. One of the most demanding goals in pulmonary tissue bioengineering is the generation of the extracellular matrix, whose proteins are essential for host-derived protection and graft homeostasis. Synthetic scaffolds can offer proper gas exchange, but due to the lack of extracellular matrix proteins, they do not provide all the requirements for effective replacement of lung function. Another major issue to be managed is the reproduction of a compact vascular flow network that is able to provide physiologic blood flow and gas exchange, thus reproducing the architectural hierarchy of the lung. One way to manage these limitations is human donor lung decellularization: this process, in fact, makes it possible to use an identical template of vascular and airway structures; however, if recellularization is not properly completed, extracellular matrix proteins will remain exposed, thus inducing a pathological reparative process in vivo, resulting in disruption of the extracellular matrix and scaffold degradation. Last but not least, the decellularization process requires human lung donors, so this technique does not solve the human organ shortage, highlighting the problem of xenogeneic scaffold use [88,89]. Recently, Shojaie et al. studied the role of pulmonary extra-cellular matrix in the differentiation process of pluripotent cells in vitro, disclosing the strong inductive capacity of the native lung matrix alone. They observed that extended culture of stem-cell-derived definitive endoderm on decellularized lung scaffolds resulted in differentiation into mature airway epithelium, presenting club cells, ciliated cells and basal cells with functional and morphological correspondences to native airways [90].

One of the most promising clinical applications of MSC technology in thoracic surgery is the minimally invasive management of post-resectional bronchopleural fistula (BPF), which is a pathological communication between the airways and the pleural cavity that may happen after major lung resections [91]. In oncologic lung resections, the incidence of BPF varies from 1% to 4%, but its mortality has been reported to range from 12.5% to 71.2%. BPF may be due to imperfect bronchial suture, obstacles to bronchial stump healing or stump impairment by remaining neoplastic cells [92]. The clinical consequence of bronchial healing failure after major lung resections may result in a life-threatening condition due to pleural empyema and ventilatory catastrophe. For the vast majority of patients developing pleural empyema, the presence or not of an active fistula makes the difference between potential recovery, chronicity or death [92]. In our previous experimental studies on preclinical large animal models, we observed a successful BPF closure by autologous bone-marrow-derived MSC bronchoscopically injected at the target bronchial stump. Fibroblast proliferation and collagenous matrix development successfully occluded BPF by tissue regeneration, thus preventing a life-threatening pleural empyema [92].

Computed tomography (CT) scanning and magnetic resonance imaging (MRI) of harvested bronchial stumps disclosed extrabronchial tissue proliferation in animals receiving MSC bronchoscopic injection, thus suggesting peribronchial tissue regeneration as one of the possible MSC-induced reparative mechanisms [92]. Stimulated by our preclinical findings and by other similar results elsewhere [93], we bronchoscopically transplanted autologous bone marrow–MSC into the bronchial stump of a male patient previously submitted to right extrapleural pneumonectomy for early-stage malignant mesothelioma and the developing small-caliber BPF [94]. Ten million autologous bone-marrow-derived MSC were inoculated into the pars membranacea of the right main bronchial stump very close to the hole of the BPF. Sixty days later, a follow-up bronchoscopy revealed full stump healing without any remaining sign of BPF, and an endobronchial biopsy revealed a hyperplastic respiratory epithelium with reduced bands of smooth muscle fibers substituted by fibroblasts [93]. The mechanism of MSC action was thought to be due to the differentiation of MSC after implantation at the target site. More recently, it has been observed that MSC act by secreting extracellular vesicles into the extracellular space. In particular, extracellular vesicles deriving from oral tissue disclose more interesting properties in repair and regeneration processes than those from all other tissues. This is probably due to their lower invasiveness and easier accessibility for sample collection [95]. Bottoni et coll. reported a bronchoscopic technique based on the injection of autologous fat tissue for treating post-resectional BPF (lipofilling). They applied this procedure to eight patients presenting BPF greater than 8 mm in diameter and observed complete BPF resolution in all treated patients [96]. Similarly, Marchioni et al. reported the effective treatment of a 7 mm BPF by means of endoscopic autologous fat implantation in two patients after right lower lobectomies [97].

With regard to the preclinical model, although extensive research has been conducted on pulmonary regeneration, the development of vital tissue organs has still not been obtained. Lung regeneration has been successfully attempted in preclinical settings by using the fetal rat lung tissue implantation model. Fetal rat lung tissue, in fact, disclosed an effective potential of growth, differentiation and proliferation by using MSC as an ideal scaffold, not only for normal lungs but also for diseased lungs. Recently, Matsumoto et coll. demonstrated that engrafted fetal lung tissue was able to create capillary and airway connections with the receiving lungs, and this process was further accelerated by corticosteroids [98].

With regard to clinical practice, recently, Martinod Martinod et al. described the use of stented heterologous aortic segment to substitute extensive tracheal defects following airway resection [99,100]. The graft was then covered by a muscle flap to boost neoangiogenesis and prevent future disruption. The authors observed cartilage and new epithelial cell growth within the grafts, thus disclosing the basic principles of tissue regeneration [99,100].

## 6. Drug Loading and Delivery

The main target of chemotherapy is to improve patient outcomes by upscaling the drug concentration in the target organs and tissues, thus boosting therapeutic efficiency and, at the same time, limiting the damage to non-neoplastic cells, reducing clinical toxicity. Nanomedicine modifies the delivery of antineoplastic drugs to the tissues, thereby considerably reducing the dose-limiting side effects while preserving or even enhancing their efficacy [101]. Polymer-based or synthetic lipid vector systems or natural carriers like extracellular vesicles, bacteria and viruses have been proposed and used as drug-delivery carriers. MSC—which have been extensively used in regenerative medicine—have been recently proposed as natural vectors for targeted activity against tumor cells, thus minimizing side effects to other non-neoplastic tissues [102,103]. New therapeutic approaches of cell-based delivery of chemotherapeutic drugs have been extensively studied thanks to the ability of MSC to move and implant into neoplastic tissues after intravenous injection. MSC exposed to high doses of some chemotherapeutic agents disclosed the property of intracellularly storing antitumor drugs and then delivering them only to neoplastic tissues without any genetic or cellular change, thus reducing cancer proliferation [104]. During the last few years, several different techniques of drug loading and delivery have been reported: immunoconjugates for linking to cancer-specific antigens, nanoparticles, genetically modified and nano-engineered stromal cells. With regard to stem cell modifications, glycoengineering protocols to produce the exhibition of non-natural azide groups on the external part of MSC—without conditioning their viability or cancer-homing properties—have been described, and nano-engineered MSC were obtained by conditioning human MSC with drug-loaded polymeric nanoparticles [105]. Nevertheless, non-modified MSC are still perhaps the most appropriate option for antineoplastic drug loading and delivery, given the fact that they easily adapt to culture environment and are readily home to neoplastic tissue when inoculated “in-vivo”. Nevertheless, although very encouraging results have been obtained by experiments on cellular lines or laboratory animals, this technique is still experimental, without any clinical application so far. On the one hand, MSC disclose encouraging perspectives because of their immunomodulatory role, the ability to release active soluble factors and to cross the blood–brain barrier, thus offering an additional potential therapeutic option for adult and pediatric brain neoplasms [106]. On the other hand, the topic of whether MSC cross-talk with the cancer microenvironment supports tumor suppression or, on the contrary, stimulates tumor growth is still unclear [107]. Further preclinical and basic science studies are therefore required before MSC can be used as drug carriers in daily clinical practice; moreover, before switching MSC use to clinical activity, international regulations need to be followed. Moreover, we might define the “drug loading and delivery” technique as a sort of universal approach to a wide variety of clinical conditions, where MSC represent just one of the possible available or potentially indicated options, both in oncologic and non-oncologic scenarios.

With regard to MSC antineoplastic activity, several hypotheses have been formulated: angiogenesis suppression, cell-cycle inhibition and antagonism of proliferation-related signaling pathways. Gemcitabine and paclitaxel have emerged as the best-loaded drugs by MSC, while although it has shown encouraging results “in-vivo” for pleural mesothelioma therapy, pemetrexed is not well internalized by MSC and so still lacks “in-vivo” confirmation.

Pleural mesothelioma represents the ideal model for drug loading and delivery in thoracic oncology: it is an uncommon, lethal asbestos-related tumor deriving from the mesothelial cells of the pleura. The tumor can arise at any place where mesothelial cells undergo a de-differentiation from mesenchymal cells, including the pericardium, peritoneum, tunica vaginalis of the ovary and testis, but the visceral and parietal pleurae remain the most involved sites [108]. A platinum-based doublet including a third-generation antifolate (pemetrexed or ralitrexed) still represents the first-line standard of care, although first-line chemo-immunotherapy has recently shown promising results, disclosing encouraging survival outcomes and better response rates for both sarcomatoid epithelioid subtypes [109]. Surgical resection may represent an effective therapeutic option in early-stage MP (stages I, II and selected stage III without nodal involvement and extra-thoracic diffusion) in addition to radiotherapy as adjuvant or symptomatic treatment [110]. Anyway, an effective second-line treatment for PM is still missing, thus representing a clinical model for a potential development of drug-loading and drug-delivery approach: we have previously demonstrated, in fact, that paclitaxel (PTX)-primed mesenchymal stromal cells successfully inhibit the in vitro proliferation of human mesothelioma cells [99]. Recently, Burgio et al. proposed the exploitation of extracellular vesicles isolated from primary cell cultures of pleural mesothelioma to modify them through post-loading techniques. They suggested that although the isolation efficiency could be lower by this technique, when compared to recombinant proteins, the final result would be far more representative of the actual secretome of mesothelioma, thus allowing an expression of surface receptors of tumoral exosomes much closer to the physiological one [111]. We report the favorable opinion recently expressed by the Italian Drug Agency on the procedure for the preparation of MSC loaded with paclitaxel in large-scale bioreactor systems and their storage before future clinical application. This new advanced medicinal therapy product has already been approved for a Phase I clinical trial on mesothelioma patients and might represent an additional option for MSC application as a drug loading and delivery system on other solid tumors for concurrent treatments associated with radiotherapy and surgery [112] (Table 2).

## 7. Conclusions

The clinical use of regenerative medicine concepts and stromal cell applications to thoracic surgical diseases are intriguing and promising fields; however, several difficulties still exist and should be clearly taken into consideration before switching experimental results into clinical practice. In particular, it should always be taken into consideration that under chronic inflammation, MSC are driven to inflammation areas and can transform in the tumor microenvironment; moreover, they can also boost tumor growth and metastasis. For this reason, the tumor tropism of MSC is an important aspect involved in tumorigenesis and not only repair or regeneration [116]. Clear warnings are needed against spectacular, anecdotal or sensational communications that jeopardize the complex areas of regenerative medicine and stromal cell technologies, making them even more risky and controversial.

## Figures and Tables

**Table 1:** Imaging techniques: advantages and disadvantages.

Imaging Technique	Advantages	Disadvantages
Optical Imaging	- Low costs - Excellent results in liver	- Some limits in extrahepatic districs
Nuclear Imaging	- Does not interfere with MSC activity - Can be effectively used in almost every district	- Needing of radioisotope labeling
Magnetic Resonance Imaging	- High-resolution images (50 μm to 300 μm) - Both types of contrast agent (PFC and SPIO) can be successfully used for MSC labelling	- Costs and dedicated MRI availability
Magnetic Particle Imaging	- Excellent image contrast, High sensitivity - High-level performance for single-cell tracking	- 200-cell detection limit “in vitro” - “In vivo” monitoring of graft clearance over 87 days

**Table 2:** MSC clinical application in thoracic surgery. AD: adipose-derived; BM: bone marrow; MSC: mesenchymal stromal cells; PRP: platelet-rich plasma.

Author	Date	Site	Cell/Source
Alvarez, PD [93]	April 2008	Trachea	AD MSC
Petrella, F [94]	January 2015	Bronchus	BM MSC
Diaz Agero, PJ [113]	January 2016	Bronchus	AD MSC
Dua, KS [83]	April 2016	Esophagus	PRP
Aho, JM [114]	October 2016	Bronchus	AD MSC seeded matrix graft
Zeng, Y [115]	October 2018	Bronchus	Umbilical cord MSC
Bottoni, E [96]	February 2021	Bronchus	Autologous fat
Marchioni, A [97]	February 2022	Bronchus	Autologous fat

## Data Availability

The data can be shared upon request.
